# Adjuvante Therapie des schweren und/oder refraktären bullösen Pemphigoids mit Immunadsorption – eine prospektive monozentrische Pilotstudie

**DOI:** 10.1111/ddg.15909_g

**Published:** 2026-04-08

**Authors:** Maike M. Holtsche, Nina van Beek, Christoph M. Hammers, Artem Vorobyev, Michael Kasperkiewicz, Nina Schumacher, Philip Muck, Enno Schmidt

**Affiliations:** ^1^ Klinik für Dermatologie Allergologie und Venerologie Universität zu Lübeck Lübeck Deutschland; ^2^ Universitätsklinik für Dermatologie und Allergologie Klinikum Oldenburg Oldenburg Deutschland; ^3^ Division of Dermatology Department of Medicine David Geffen School of Medicine at University of California Los Angeles Los Angeles USA; ^4^ Medizinische Klinik I Universität zu Lübeck Lübeck Deutschland; ^5^ Lübecker Institut für experimentelle Dermatologie Universität zu Lübeck Lübeck Deutschland

**Keywords:** BP180, blasenbildende Autoimmundermatosen, bullöses Pemphigoid, Immunadsorption, autoimmune blistering disease, BP180, bullous pemphigoid, immunoadsorption

## Abstract

**Hintergrund und Zielsetzung:**

Das bullöse Pemphigoid (BP) ist die häufigste blasenbildende Autoimmunerkrankung in der westlichen Welt. Während die Mehrheit der Patienten mit BP durch die langfristige Anwendung von Kortikosteroiden mit oder ohne Immunmodulatoren/Immunsuppressiva Remissionen erreicht, empfehlen nationale und internationale Leitlinien bei refraktären Erkrankungen die adjuvante Immunadsorption (IA). Ziel dieser Studie war es, die Sicherheit und Wirksamkeit der IA bei schwer betroffenen Patienten und/oder refraktärem BP zu untersuchen.

**Patienten und Methodik:**

Zehn Patienten mit BP (3 Frauen, 7 Männer; Durchschnittsalter 69,7 Jahre; 52–81 Jahre) wurden an drei aufeinanderfolgenden Tagen mit IA (LigaSorb^®^, Fresenius Medical Care) in Kombination mit oralem Prednisolon (0,5 mg/kg KG/Tag, ausschleichend), Dapson (1,5 mg/kg KG/Tag) und Mometasonfuroat‐Salbe behandelt.

**Ergebnisse:**

Innerhalb von 2 und 6 Monaten nach IA zeigten 50% beziehungsweise 90% der Patienten eine vollständige Remission unter der Therapie. Bei allen Patienten sanken die Serum‐Anti‐BP180‐IgG‐Spiegel unmittelbar nach der dritten IA um durchschnittlich 89% und 4 Wochen später um 73%. Während der 12‐monatigen Nachbeobachtungszeit traten insgesamt 56 unerwünschte Ereignisse (UE) auf. Die Mehrzahl der unerwünschten Ereignisse hatte den Schweregrad 2 (50%), 15 unerwünschte Ereignisse wurden als schwerwiegend (Grad 3–4) eingestuft.

**Schlussfolgerungen:**

Die IA kann als relativ sichere und wirksame adjuvante Therapie für Patienten mit schwerem oder refraktärem BP angesehen werden.

## EINLEITUNG

Blasenbildende Autoimmunerkrankungen (*autoimmune blistering diseases*, AIBD) werden durch Autoantikörper gegen Strukturproteine der Epidermis oder der dermoepidermalen Junktionszone verursacht.^1^, ^2^ Mit einer Inzidenz von etwa 20 Patienten pro Million Einwohner und Jahr ist das bullöse Pemphigoid (BP) die häufigste AIBD in Mittel‐ und Nordeuropa sowie in Nordamerika.^3^, ^4^, ^5^, ^6^ Die Zielantigene des BP sind zwei Strukturproteine der dermoepidermalen Junktionszone, BP180, auch als Typ‐XVII‐Kollagen oder BPAG2 bezeichnet, und BP230 (BPAG1).^7^ Der Nachweis von gewebegebundenem IgG und Komplement C3 in der direkten Immunfluoreszenz (IF) ist der diagnostische Goldstandard. Zirkulierende Autoantikörper können mittels indirekter IF auf humaner Spalthaut sowie durch ELISA mit rekombinanten Formen der beiden Zielantigene und die auf indirekter IF basierte BIOCHIP^®^‐Technik nachgewiesen werden.^7^, ^8^, ^9^ Die NC16A‐Domäne von BP180 wurde als immundominante Region ermittelt.^10^, ^11^

An BP180 gebundene Autoantikörper führen über Komplementaktivierung, die Freisetzung proinflammatorischer Mediatoren und schließlich spezifischer Proteasen zur dermoepidermalen Spaltbildung.^12^, ^13^ (Ref 41) Die aktuellen europäischen und deutschen S2k‐Leitlinien zur Behandlung des BP empfehlen die Behandlung mit topischen und systemischen Kortikosteroiden, Dapson, Doxycyclin, Methotrexat, Azathioprin, Mycophenolatmofetil und Omalizumab. Bei Patienten mit refraktären Erkrankungen werden adjuvant der Anti‐CD20‐Antikörper Rituximab, hochdosierte intravenöse Immunglobuline, Dupilumab und Immunadsorption (IA) empfohlen.^14^, ^15^

Aufgrund der schweren und häufigen Nebenwirkungen systemischer Kortikosteroide, einschließlich der erhöhten Sterblichkeit bei älteren BP‐Patienten, werden kortikosteroidsparende Therapien eingesetzt. Da die Anti‐BP180‐Autoantikörperspiegel mit der Krankheitsaktivität korrelieren und die pathogene Wirkung von Anti‐BP180‐IgG in vitro und in vivo eindeutig nachgewiesen wurde,^10^, ^16^, ^17^, ^18^ scheint die Entfernung von Autoantikörpern aus dem Blutkreislauf eine rationale Behandlungsoption zu sein. Die IA ist ein Hämoadsorptionsverfahren zur selektiven Entfernung von Antikörpern aus dem Plasma. Der Vorteil gegenüber der Plasmapherese liegt in der relativen Selektivität, dem Verzicht auf die Substitution mit Humanalbumin oder gefrorenem Frischplasma und der zwei‐ bis dreifach höheren Plasmamenge, die pro Sitzung verarbeitet werden kann. Es wird vermutet, dass die IA zur Verschiebung pathogener gewebegebundener IgG‐Autoantikörper zurück in den Kreislauf führt.^19^ Häufige Nebenwirkungen sind eine Citrat‐induzierte Hypokalzämie, Hypotonie und Infektionen durch den venösen Zugang.^20^ Die IA wurde bereits erfolgreich zur Behandlung verschiedener neurologischer, kardiologischer und rheumatologischer Erkrankungen sowie in der Nephrologie, Hämatologie und Transplantationsmedizin eingesetzt.^21^ Fallserien über die erfolgreiche Anwendung der IA wurden auch bei Patienten mit refraktärer atopischer Dermatitis und Pemphigus berichtet.^22^, ^23^, ^24^, ^25^, ^26^

Kürzlich zeigte eine multizentrische randomisierte kontrollierte Studie bei Pemphigus vulgaris/foliaceus eine signifikant niedrigere kumulative Prednisolondosis in der Gruppe mit IA plus Standardtherapie im Vergleich zu der Gruppe, die lediglich Standardtherapie erhielt. Der primäre Endpunkt, also die Zeit bis zur vollständigen Remission unter Therapie, unterschied sich jedoch nicht signifikant zwischen den Gruppen.^27^ In der vorliegenden Studie wurden zehn Patienten mit schwerem und/oder refraktärem BP mit IA in Kombination mit der Standardtherapie des BP behandelt, das heißt mit ausschleichend verabreichtem Prednisolon (0,5 mg/kg Körpergewicht [KG]/Tag p.o.), läsional applizierter Mometasonfuroat‐Salbe und Dapson.

## PATIENTEN UND METHODIK

### Patienten

Die Patienten wurden zwischen Juni 2018 und Oktober 2021 in der Klinik für Dermatologie der Universität zu Lübeck mit adjuvanter IA behandelt. Bei der großen Mehrheit der Patienten wurde die IA vor dem Ausbruch der SARS‐CoV2‐Pandemie in Deutschland durchgeführt, nur die IA von Patient 10 fiel in den Zeitraum der Pandemie. Es wurden vier Patienten mit schwerem BP und sechs Patienten, die auf die Erstlinientherapie refraktär waren, eingeschlossen. Die Gruppe der therapierefraktären Patienten wurde zuvor mit Prednisolon, Dapson, i.v. Dexamethason‐Pulsen und Azathioprin behandelt, wie in Tabelle 1 dargestellt. Gemäß den Leitlinien der *Deutschen Dermatologischen Gesellschaft* wurde eine schwere Erkrankung als mehr als 30% der Körperoberfläche definiert.^15^ Die Diagnose basierte auf einer positiven direkten IF und zirkulierenden Anti‐BP180‐IgG mittels ELISA (Euroimmun, Lübeck, Deutschland).^11^ Ausschlusskriterien waren akute und chronische Infektionen, Gerinnungsstörungen, schwere kardiovaskuläre Erkrankungen, aktive Malignität, Verwendung von ACE‐Hemmern, Schwangerschaft und Stillzeit sowie Allergien gegen Studienmedikamente. Die Studie wurde in Übereinstimmung mit der Deklaration von Helsinki durchgeführt und von der Ethikkommission der Universität Lübeck genehmigt (17‐185). Drei Frauen und sieben Männer wurden nach schriftlicher Einwilligung in die Studie eingeschlossen. Das Durchschnittsalter betrug 69,7 Jahre und reichte von 52–81 Jahren. Die Patienten litten unter einer starken Beeinträchtigung der Lebensqualität mit einem mittleren DLQI (*Dermatology Life Quality Index*) von 19,1/30 (2–27) zum Zeitpunkt des Studieneinschlusses (Tabelle 1). Die meisten Patienten befanden sich zu diesem Zeitpunkt in einem guten Allgemeinzustand, gemessen am ECOG‐Index (Index der *Eastern Co‐operative of Oncology Group*) (Tabelle 1).

**TABELLE 1:** Patientencharakteristika zu Beginn der Studie und bisherige Therapien.

Patient	Alter (Jahre)	Sex	Krankheitsaktivität	Krankheitsdauer (Monate)	Bisherige Therapien	ECOG	DLQI
1	72	F	Schwer	0	Keine	2	12
2	81	M	Schwer	0	Keine	1	13
3	79	F	Refraktär	3	PRE, DAP, DEX	1	14
4	65	M	Refraktär	4	PRE, DAP, DEX	1	27
5	74	M	Refraktär	2	PRE, DAP	3	21
6	57	F	Schwer	0	Keine	1	27
7	80	M	Schwer	0	Keine	1	2
8	58	M	Refraktär	60	AZA, DEX, DAP, PRE	1	26
9	79	M	Refraktär	14	PRE, DAP	0	23
10	52	M	Refraktär	60	PRE, DAP	0	26

*Abk*.: ECOG, *Index of Eastern Co‐operative of Oncology Group*; DLQI, *Dermatology Life Quality Index*; PRE, Prednisolon; DAP, Dapson; DEX, intravenöse Dexamethason‐Pulse (100 mg/Tag an drei aufeinanderfolgenden Tagen); AZA, Azathioprin

### Behandlungsprotokoll

**ABBILDUNG 1:**
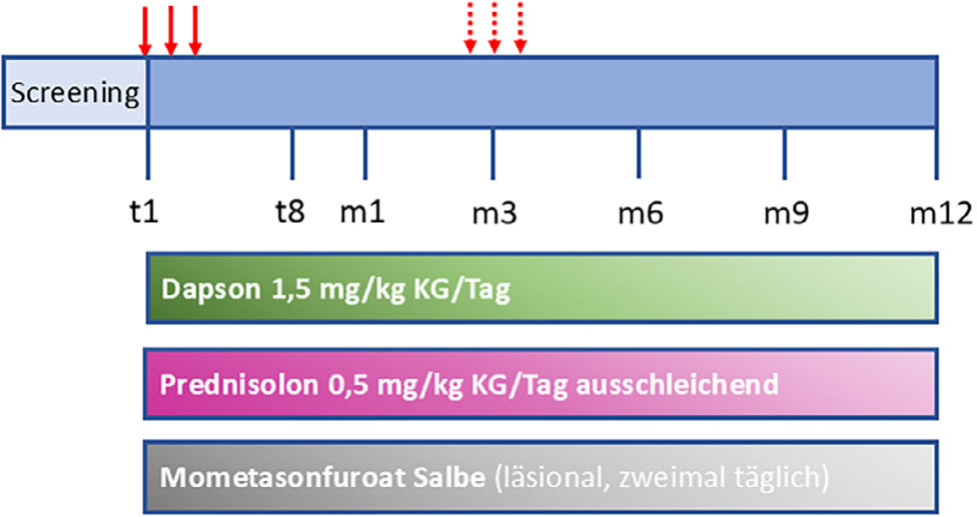
Behandlungsprotokoll. Die Immunadsorption wurde an drei aufeinanderfolgenden Tagen durchgeführt (rote Pfeile). Falls aufgrund eines Rückfalls erforderlich, wurde sie zu einem individuellen Zeitpunkt wiederholt (rot gestrichelte Pfeile). Zusätzlich erhielten alle Patienten Dapson, Prednisolon und läsional applizierte Mometasonfuroat Salbe. *Abk*.: T, Tag; m, Monat; KG, Körpergewicht.

Die IA wurde in der Abteilung für Nephrologie der Klinik für Innere Medizin I der Universität zu Lübeck durchgeführt. Bei allen Patienten erfolgte der venöse Zugang über einen temporären, nicht getunnelten zentralen Dialysekatheter (Shaldon) der Vena jugularis interna und die Plasmaseparation wurde mit einem Plasmaseparationsgerät (COM.TEC^®^, Fresenius Kabi AG, Bad Homburg, Deutschland) durchgeführt. Zur Antikoagulation erhielten die Patienten 5000 IE Heparin‐Natrium sowie Citrat‐Dextrose, Formel A (ACD‐A, Fresenius HemoCare Austria GmbH, Eugendorf, Österreich). Das Plasma wurde mit Hilfe eines Plasmaflussmonitors (ADAsorb^®^, Medicap Clinic GmbH, Ulrichstein, Deutschland) mit einer Rate von 25 bis 40 ml/min durch eine Protein‐A‐Säule (Ligasorb^®^, Fresenius Medical Care, Bad Homburg, Deutschland) geleitet. Die Säule wurde mit Plasma beladen, die gebundenen Antikörper wurden eluiert (Elutionslösung ADAsorb pH2,2, Serag Wiessner GmbH, Naila, Deutschland) und die Säule wurde anschließend rekalibriert (multiPlus 2K, Fresenius Medical Care). Die IA wurde an drei aufeinanderfolgenden Tagen (Tag 1–3) durchgeführt. Im Falle eines Rezidivs mit hohen Autoantikörperspiegeln konnte während der gesamten Studiendauer jederzeit ein weiterer dreitägiger IA‐Zyklus durchgeführt werden. Wie in den deutschen Leitlinien empfohlen, erhielten die Patienten eine Standardtherapie mit oralem Prednisolon (0,5 mg/kg KG/Tag, ausschleichend), Dapson (1,5 mg/kg KG/Tag) und, abweichend von der Leitlinie, Mometasonfuroat‐Salbe (läsionale Anwendung, zweimal täglich) (Abbildung 1).^15^ Die Prednisolon‐Dosis wurde so lange beibehalten, bis sich eine Woche lang keine neuen Blasen bildeten. Dann wurde Prednisolon um 25% reduziert, wenn weiterhin keine neuen Läsionen mehr auftraten, folgten wöchentliche Reduktionen, zunächst um 25% (auf 0,25 mg/kg KG/Tag), dann in 5‐mg‐Schritten, bis 10 mg/Tag erreicht waren, dann in 2,5‐mg‐Schritten bis 5 mg/Tag. Die nächsten Reduktionsschritte folgten in zweiwöchigen Abständen auf 2,5 mg/Tag, 2,5 mg jeden zweiten Tag und Absetzen. Dapson 1,5 mg/kg KG/Tag wurde in einer morgendlichen Dosis verabreicht, bis das Prednisolon abgesetzt wurde. Nachdem Prednisolon abgesetzt wurde und einen Monat lang keine neuen Blasen aufgetreten waren, wurde Dapson in monatlichen Abständen um 25 mg reduziert und schließlich ebenfalls abgesetzt. Mometasonfuroat‐Salbe wurde anfangs zweimal täglich auf die betroffenen Hautstellen aufgetragen und abgesetzt, wenn über einen Zeitraum von einer Woche keine neuen Läsionen auftraten. Außerdem wurden die Blasen steril geöffnet und, falls erforderlich, mit 0,5%iger Pyoktanin‐Lösung behandelt. Die Nachuntersuchungen umfassten eine körperliche Untersuchung mit Bewertung des BPDAI (*Bullous pemphigoid disease area index*) einschließlich Pruritus‐Score (0: kein Juckreiz, 10: leichter Juckreiz, 20: mäßiger Juckreiz, 30: starker Juckreiz), Routinelaborparametern und zirkulierenden Anti‐BP180‐NC16A‐IgG und Anti‐BP230‐IgG mittels ELISA (Euroimmun) sowie den DLQI. Der Karnofsky‐Index wurde beim Screening und beim letzten Studienbesuch bestimmt. Das Outcome wurde kategorisiert in Komplettremission unter Therapie (Abheilung aller Läsionen während der Behandlung), Teilremission (Abheilung von > 50% der Läsionen) und Rezidiv (3 oder mehr neue Läsionen im letzten Monat, die nicht innerhalb einer Woche bei einem Patienten abheilen, der zuvor eine Komplettremission erreicht hatte).

### Statistische Analyse

Die p‐Werte wurden mit dem Wilcoxon Signed Rank Test ermittelt. Das Programm GraphPad Prism (Version 7, GraphPad Software, San Diego, CA, USA) wurde für die statistische Datenanalyse verwendet. Das Signifikanzniveau wurde auf α = 0,05 festgelegt.

## ERGEBNISSE

### Klinischer Krankheitsverlauf

**ABBILDUNG 2:**
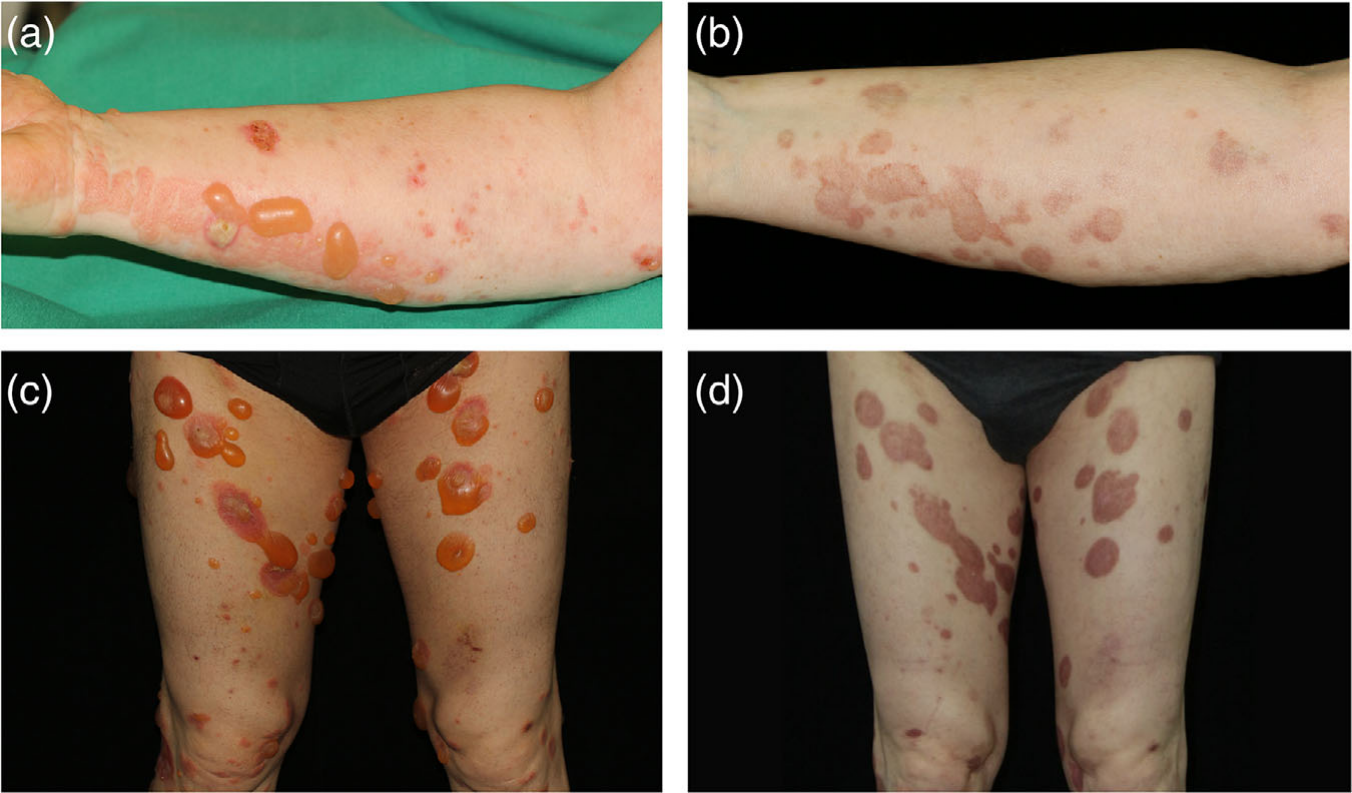
Klinischer Verlauf. (a) Pralle Blasen auf erythematöser Haut bei Patient 6 u (c) Patient 8 vor dem ersten Zyklus der Immunadsorption. (b) Reepithelisierung mit postinflammatorischer Hyperpigmentierung vier Wochen nach dem ersten Zyklus der Immunadsorption bei Patient 6 und (d) Patient 8.

**ABBILDUNG 3:**
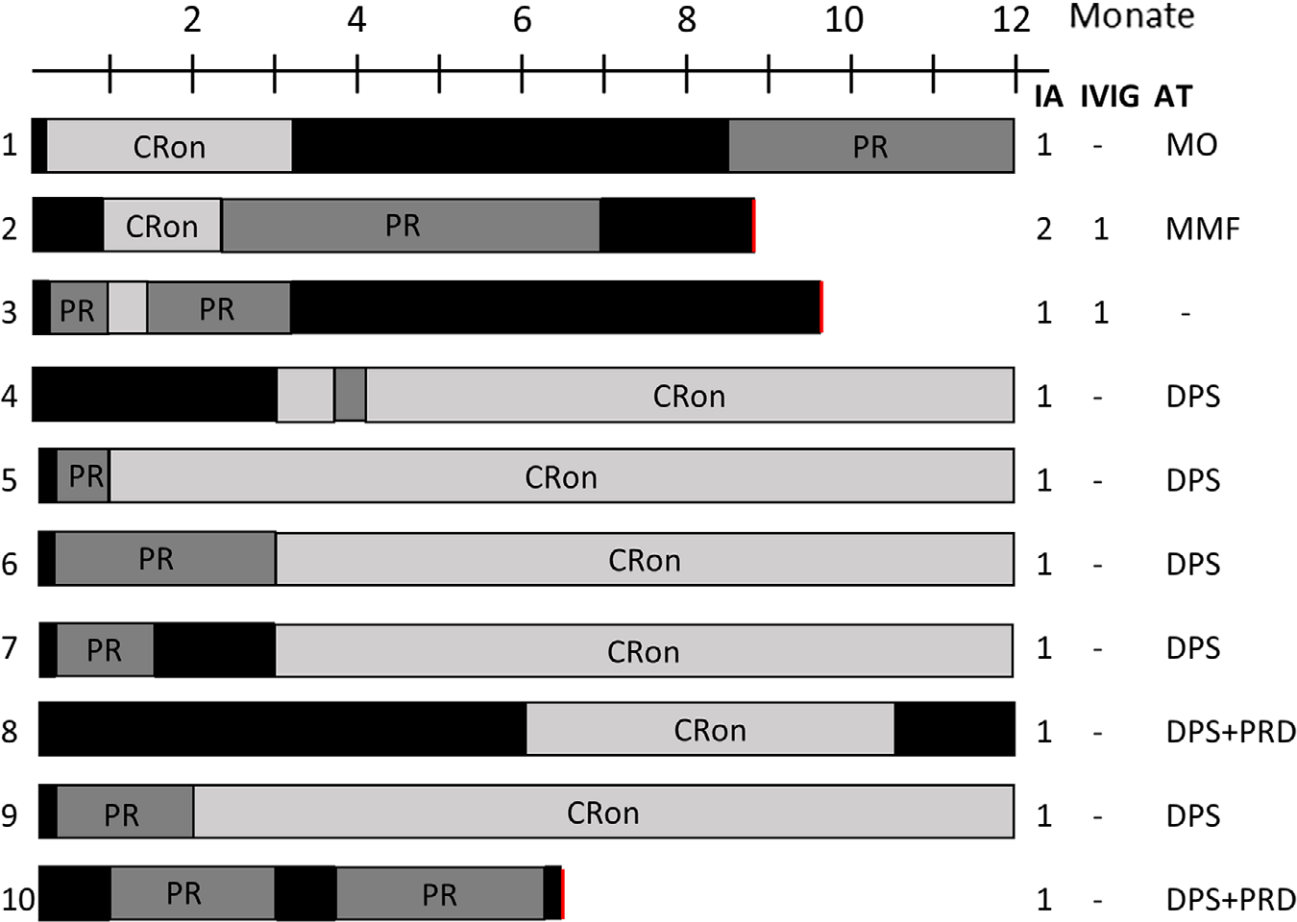
Krankheitsverläufe und Behandlungsdetails. Schwarzes Kästchen, aktive Erkrankung oder Rückfall. *Abk*.: PR, partielle Remission; CRon, komplette Remission unter Therapie; roter Balken, Ausschluss aus der Studie; IA, Immunadsorptionszyklus (3 Tage); AT, aktuelle Therapie am Studienende; DPS, Dapson; MMF, Mycophenolatmofetil; MO, Mometasonfuroat‐Salbe; PRD, Prednisolon.

**ABBILDUNG 4:**
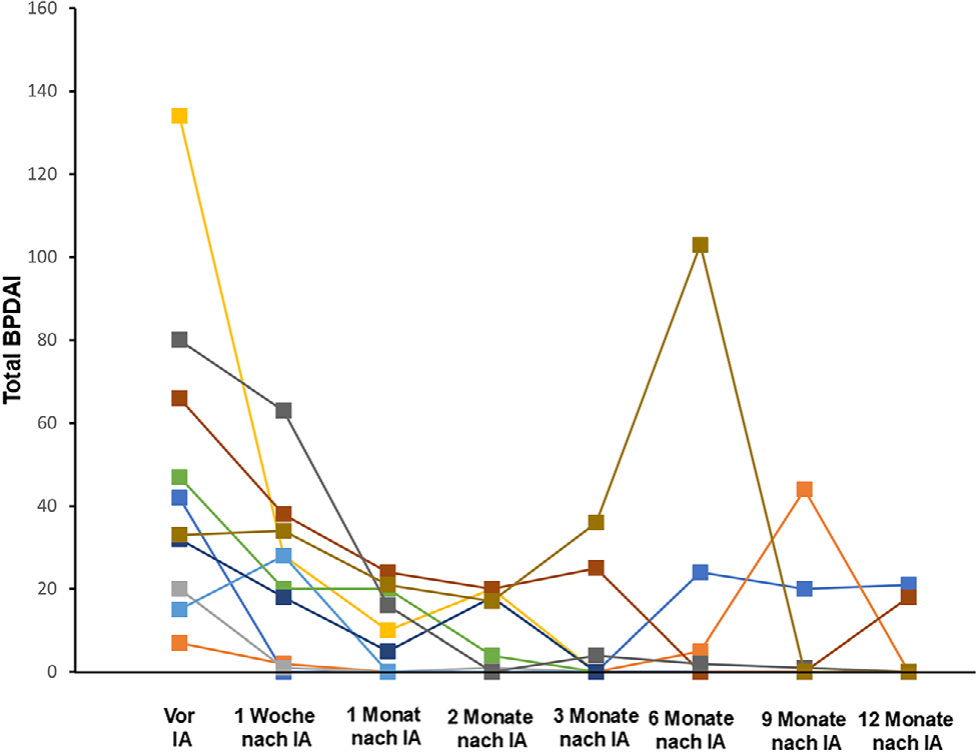
Gesamt‐BPDAI‐Werte im Krankheitsverlauf. Jeder Patient ist durch eine Farbe dargestellt. *Abk*.: IA, Immunadsorption; BPDAI, bullous pemphigoid disease area index; IVIG, hochdosierte intravenöse Immunglobuline

Innerhalb von 2 Monaten nach der IA erreichten 50% der Patienten unter der Therapie eine vollständige Remission, und innerhalb von 6 Monaten stieg diese Zahl auf 90% (Abbildungen 2, 3). Die Krankheitsaktivität, gemessen am Gesamt‐BPDAI, ging bereits eine Woche nach der IA im Vergleich zum Ausgangswert deutlich zurück (p = 0,0137). Zu späteren Zeitpunkten, das heißt 1 (p = 0,002), 2 (p = 0,002), 3 (p = 0,0039), 6 (p = 0,0488), 9 (p = 0,0273) und 12 Monate (p = 0,002) nach der ersten IA war der BPDAI ebenfalls signifikant niedriger im Vergleich zum Ausgangswert (Abbildung 4). Prednisolon wurde ausgeschlichen und nach durchschnittlich 5,5 Monaten vollständig abgesetzt. Innerhalb der Nachbeobachtungszeit von 12 Monaten traten bei sechs Patienten Rezidive auf. Gemäß dem Protokoll war es möglich, im Falle eines Rezidivs einen zweiten IA‐Zyklus durchzuführen, was bei einem Patienten (Patient 2) tatsächlich erfolgte. Sieben der zehn Patienten wurden bis zum Ende der Studie (12 Monate) nachbeobachtet, zwei Patienten schieden wegen eines zweiten Rezidivs (nach sechs beziehungsweise 9 Monaten) und einer wegen fortgesetztem Krankenhausaufenthalt aus anderen Gründen (nach 9 Monaten) aus. Keiner der zehn Patienten verstarb während der Nachbeobachtungszeit von einem Jahr. Die Krankheitsverläufe und die BP‐Medikation sind in Abbildung 3 dargestellt. Die Lebensqualität hat sich bei allen Patienten 1 Monat nach der IA im Vergleich zum Ausgangswert signifikant verbessert (p = 0,0039). Ebenso ging der Pruritus bereits eine Woche nach der IA signifikant von einem Wert von 10,0 auf 0 zurück (p = 0,0156). Alle DLQI‐ und Pruritus‐Scores sind in der ergänzenden Tabelle S1 im Online‐Supplement aufgeführt.

### Komedikation mit Prednisolon und Dapson

Prednisolon wurde zunächst in einer Dosis von 0,5 mg/kg KG/Tag verabreicht und dann, wie im Abschnitt Patienten und Methodik beschrieben, schrittweise reduziert. Bei acht Patienten konnte Prednisolon abgesetzt werden; drei dieser Patienten erlitten ein Rezidiv, nachdem Prednisolon vollständig abgesetzt worden war, bei einem von ihnen wurde Prednisolon wieder eingeleitet. Im Durchschnitt wurde Prednisolon nach 5,5 Monaten abgesetzt (3 Monate bis anhaltend). Alle Patienten erhielten 6 Monate nach Beginn der Therapie weniger als 7,5 mg/Tag. Bei sieben Patienten wurde die Behandlung mit Dapson über das Ende der Studie hinaus fortgesetzt. Bei den verbleibenden drei Patienten wurde es aufgrund von Anämie/Dyspnoe, wie im Abschnitt Unerwünschte Ereignisse beschrieben, abgesetzt.

### Autoantikörperspiegel

**ABBILDUNG 5:**
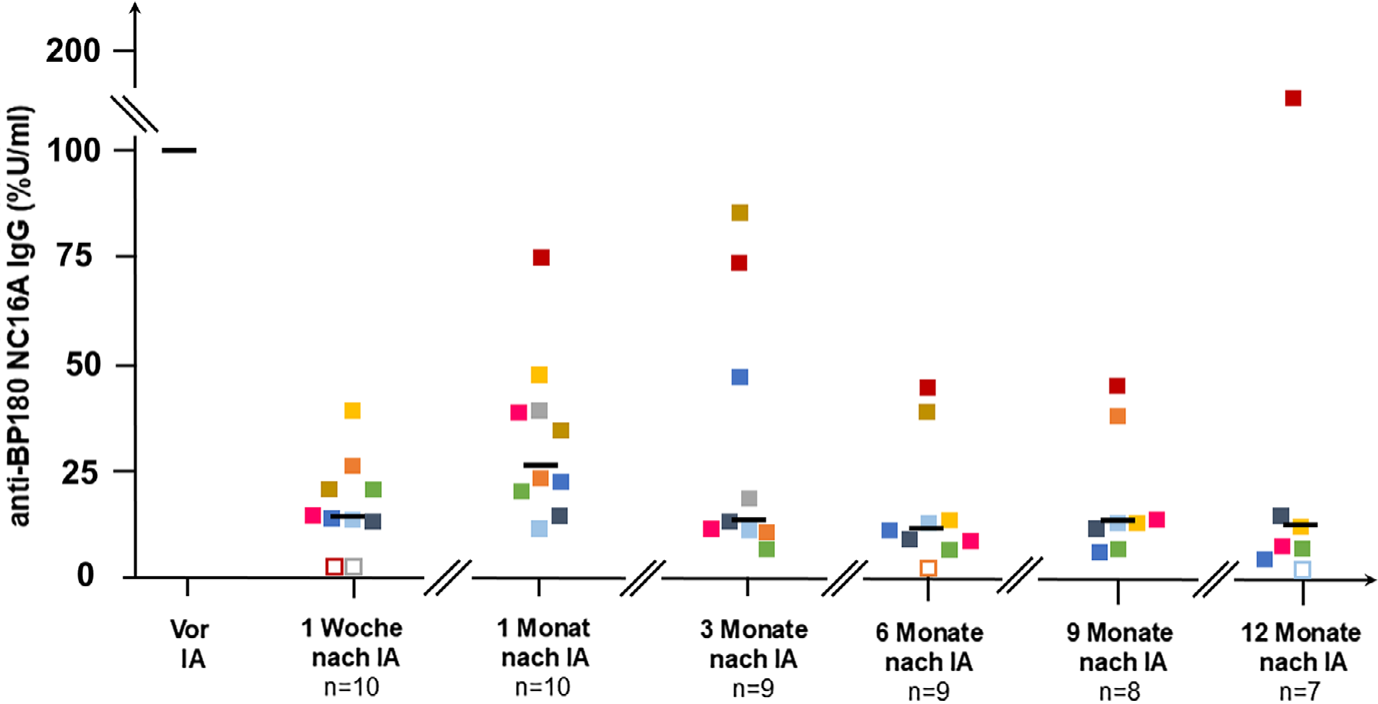
Serumspiegel von Anti‐BP180‐NC16A‐IgG als Prozentsatz der Ausgangswerte mit Median im Krankheitsverlauf. Positive Werte (> 20 U/ml) wurden durch ausgefüllte Kästchen dargestellt, negative Werte durch leere Kästchen. Jeder Patient ist durch eine bestimmte Farbe dargestellt. Der Median der Anti‐BP180‐IgG‐Werte ist durch schwarze Balken dargestellt. *Abk*.: IA, Immunadsorption.

Bei allen Patienten sanken die Serumspiegel der Autoantikörper gegen BP180 nach der IA signifikant, und zwar um durchschnittlich 89% im Vergleich zum Ausgangswert unmittelbar nach der dritten Sitzung der IA (p = 0,004) und um 73% 4 Wochen später (p = 0,002). Auch 3, 6 und 9 Monate nach der IA waren die Anti‐BP180‐Antikörperspiegel im Vergleich zum Ausgangswert deutlich erniedrigt (p = 0,004, p = 0,004 beziehungsweise p = 0,008). Bei den Patienten 3 und 8 waren unmittelbar nach der 3. IA keine Anti‐BP180‐IgG im Serum mehr nachweisbar (Abbildung 5). Die Anti‐BP230‐IgG‐Serumspiegel nahmen 4 Wochen (p = 0,03) und 6 Monate (p = 0,03) nach der IA deutlich ab. Die Anti‐BP180‐ und Anti‐BP230‐IgG‐Serumspiegel sind in den Tabellen S2 und S3 im Online‐Supplement aufgeführt.

### Unerwünschte Ereignisse

Während der zwölfmonatigen Nachbeobachtungszeit traten insgesamt 56 unerwünschte Ereignisse (UE) auf (1 bis 12 Ereignisse/Patient). Der Schweregrad wurde anhand der *Common Terminology Criteria for Adverse Events V3.0* klassifiziert. Die Mehrzahl der UE waren Grad 2 (50%), die anderen wurden als Grad 1 (34%), Grad 3 (13%) und Grad 4 (3%) eingestuft. Anämie und/oder Dyspnoe wurden 10‐mal beobachtet und überwiegend als Grad 1 oder 2 bewertet (90%). Bei Patient 1 wurde Dapson nach dem ersten Auftreten einer Anämie abgesetzt und Erythrozytenkonzentrate verabreicht. Als Dapson mit einer niedrigeren Dosis wieder fortgesetzt wurde, trat die Anämie trotz normaler Glukose‐6‐Phosphat‐Dehydrogenase kurz darauf erneut auf, und Dapson wurde vollständig abgesetzt. Bei den Patienten 2 und 3 wurde Dapson wegen einer Anämie mit Hämoglobinwerten unter 8,6 g/dl trotz normaler Glukose‐6‐Phosphat‐Dehydrogenase abgesetzt und wegen der anhaltenden Anämie nicht wieder eingeleitet. Bei den Patienten 5 und 7 wurde Dapson reduziert, was zur Normalisierung der Hämoglobinwerte führte. Fünfzehn UE wurden als schwere UE eingestuft, von denen acht (Angina pectoris, Ekzem, BP‐Rezidiv, hypertensive Krise und unklare Infektion) möglicherweise mit der Studienmedikation zusammenhingen, während bei sieben (Gicht, Bronchialkarzinom, Norovirus‐Infektion, Bursitis olecrani, Beckenringfraktur, Aufenthalt Intensivstation, Sturz) ein Zusammenhang mit der Studienmedikation unwahrscheinlich erschien. Das mittlere Intervall zwischen dem letzten Tag der IA und dem Auftreten schwerer UE betrug 84 Tage (26‐277 Tage). Die meisten schweren UE (7(46%)) traten bei einem Patienten (Patient 3) auf. Alle UE sind in der ergänzenden Tabelle S4 im Online‐Supplement aufgeführt.

## DISKUSSION

Seit der Einführung der IA im Jahr 1969 wurde dieses Verfahren auch bei schweren und/oder refraktären Fällen von AIBD, einschließlich BP, eingesetzt. Bisher wurde über 35 Patienten mit BP, die mit adjuvanter IA behandelt wurden, berichtet, die meisten von ihnen als Fallberichte oder kleine Fallserien.^28^, ^29^, ^30^, ^31^, ^32^, ^33^, ^34^ Es liegt keine randomisierte, kontrollierte Studie zur IA bei BP‐Patienten vor. Im Jahr 1997 behandelten Ino et al. die ersten beiden BP‐Patienten mit der regenerierbaren Dextransulfat‐Zellulosesäule (Selesorb, Kanegafuchi Chemical, Osaka, Japan) in Kombination mit Kortikosteroiden beziehungsweise Kortikosteroiden plus Dapson.^31^ Ein Patient erhielt sechs Apherese‐Behandlungen über 2 Wochen, welche zu einer klinischen Remission führten. Bei dem zweiten Patienten wurde die IA viermal innerhalb von 2 Wochen durchgeführt und anschließend viermal wiederholt, als 6 Wochen nach dem ersten Behandlungszyklus ein Rezidiv der Hautläsionen auftrat. Eine vollständige Remission wurde bei keinem von ihnen erreicht. Herrero‐González et al. berichteten über die Anwendung der IA bei zwei BP‐Patienten, die refraktär gegenüber der Erstlinienbehandlung waren und bei denen die nicht regenerierbare Einweg‐Tryptophan‐gebundene Matrix Immusorba TR‐350^®^ (Asahi Medical, Tokio, Japan) eingesetzt wurde.^32^ Es wurden zwei IA‐Sitzungen an aufeinanderfolgenden Tagen durchgeführt; einem Patienten wurde zusätzlich ein Zyklus intravenöses Immunglobulin (IVIG) zur Infektionsprophylaxe verabreicht. Beide Patienten erreichten nach sechs Wochen eine vollständige Remission unter Therapie. Beide Patienten erreichten nach 6 Wochen eine vollständige Remission unter Therapie. Im Jahr 2012 beschrieben Müller et al. einen BP‐Patienten, der auf die Zweitlinienbehandlung mit IVIG nicht ansprach.^34^ Es wurden zwei Zyklen IA an drei aufeinanderfolgenden Tagen im Abstand von 14 Tagen mit der regenerierbaren, wiederverwendbaren Protein‐A‐Säule Immunosorba^®^ (Fresenius Medical Care) durchgeführt, was zur Teilremission unter Therapie führte. In der Fallserie von Kasperkiewicz et al. mit sieben BP‐Patienten wurde die IA an drei aufeinanderfolgenden Tagen (Immunosorba^®^, Fresenius Medical Care) in Kombination mit Prednisolon 0,25 mg/kg KG/Tag, Dapson 1,0–1,5 mg/kg KG/Tag und Clobetasolpropionat 0,05% Salbe zweimal täglich angewendet.^29^ Alle Patienten erreichten eine vollständige Remission unter Therapie 1 bis 3 Monate nach der IA, sechs der sieben Patienten zeigten ein langfristiges Ansprechen. Unter Verwendung desselben Adsorbers wurde die IA bei drei Patienten sieben‐ bis zehnmal über einen Zeitraum von 4 bis 9 Wochen in Kombination mit Rituximab und konventioneller Immunsuppression durchgeführt.^33^ Bei zwei Patienten kam es zu einer vollständigen Remission, während ein Patient eine Teilremission erreichte. Die bisher größte Fallserie zur IA bei BP‐Patienten von Hübner et al. umfasste 20 Patienten.^28^

Die IA wurde an drei aufeinanderfolgenden Tagen durchgeführt (ein Zyklus) (Immunosorba^®^, Fresenius Medical Care), zusätzlich zu Dapson, Prednisolon und topischer Clobetasolpropionat 0,05% Salbe. Zwei der 18 Patienten benötigten zusätzliche IA‐Zyklen. Einer der Patienten erhielt einen, ein weiterer drei zusätzliche Zyklen im Abstand von 2 Wochen. Alle Patienten zeigten ein schnelles und anhaltendes Ansprechen, Anti‐BP180‐Serum‐IgG sank nach einem Monat auf 26% des Ausgangswertes.^28^ Im Gegensatz zu dieser Studie wurden in der vorliegenden Studie alle Patienten prospektiv nach einem standardisierten Protokoll behandelt.

Wir behandelten zehn BP‐Patienten mit einem Zyklus IA an drei aufeinanderfolgenden Tagen sowie Dapson (1,5 mg/kg KG/Tag), Prednisolon (0,5 mg/kg KG/Tag) und zweimal täglich Mometasonfuroat Salbe läsional. Prednisolon wurde rasch ausgeschlichen. Die positiven Ergebnisse früherer Fallserien, die eindeutige Rolle von Autoantikörpern in der Pathogenese des BP und der Bedarf an neuen therapeutischen Optionen für schwere und/oder refraktäre BP‐Patienten stellten die Rationale für unsere Studie dar. Insbesondere da Kortikosteroide, die nach wie vor die Grundsäule der BP‐Behandlung bilden, mit schwerwiegenden Nebenwirkungen und einer erhöhten Sterblichkeit einhergehen,^35^ gibt es einen großen medizinischen Bedarf für Therapien, die die kumulative Dosis von Kortikosteroiden und damit die Anzahl und den Schweregrad von Nebenwirkungen reduzieren können. In diesem Sinne zeigte eine kürzlich durchgeführte randomisierte kontrollierte Studie zur adjuvanten IA bei Pemphigus vulgaris/foliaceus eine signifikante Verringerung der kumulativen Kortikosteroiddosis in der Gruppe mit IA plus bester medizinischer Behandlung im Vergleich zu Patienten, die nur die beste medizinische Behandlung erhielten.^27^ Generell kann zwischen wiederverwendbaren und Einweg‐, sowie regenerierbaren und nicht regenerierbaren Säulen unterschieden werden. Nicht regenerierbare Säulen enthalten die Liganden Phenylalanin, Tryptophan, Dextransulfat und hydrophobe Aminosäure und können nur in einer einzigen Sitzung verwendet werden, bis ihre Kapazität erschöpft ist.

Im Gegensatz dazu werden regenerierbare Adsorber paarweise verwendet. Während die eine Säule regeneriert wird, kann die andere mit Plasma beladen werden, so dass ein kontinuierlicher Aphereseprozess möglich ist. Protein A, das synthetische Peptid PGAM146 oder gegen humanes Ig gerichtete Schaf‐Antikörper werden als Liganden in diesen regenerierbaren, wiederverwendbaren Adsorbern eingesetzt, die für bis zu 10–20 Verfahren bei ein und demselben Patienten wiederverwendet werden können.^36^ Im Vergleich zur Studie von Hübner et al., in der eine wiederverwendbare, regenerierbare Protein‐A‐Säule (Immunosorba^®^, Fresenius Medical Care) verwendet wurde,^28^ wurde in unserer aktuellen Studie eine regenerierbare Einweg Protein‐A‐Säule (Ligasorb^®^, Fresenius Medical Care) verwendet. So wurde für jedes IA‐Verfahren eine Säule verwendet, was die Kosten für das Säulenmaterial um 25% im Vergleich zur Verwendung des Immunosorba^®^‐Säulenpaars in unserer früheren Studie reduzierte.^28^ In der vorliegenden Studie zeigten 50% der Patienten innerhalb von 2 Monaten nach der IA eine vollständige Remission, was mit den Ergebnissen von Hübner et al. vergleichbar ist, die bei 42% der Patienten 1 Monat nach der IA eine vollständige Remission erzielten.^28^ In der letztgenannten Fallserie war die IA vor allem bei Patienten erfolgreich, die auf die vorherige Behandlung nicht ansprachen, während in unserer Studie sowohl refraktäre als auch behandlungsnaive schwer betroffene Patienten gut ansprachen. Im Gegensatz zur Studie von Hübner et al. wurden in der vorliegenden Studie auch Juckreiz und Lebensqualität während des Krankheitsverlaufs quantifiziert. Bemerkenswerterweise verbesserte sich die Lebensqualität bereits 1 Monat nach der IA signifikant von 19,1 ± 8,0 (Mittelwert ± Standardabweichung [SD]) auf 6,8 ± 6,3 (p = 0,0039). Ebenso reduzierte sich der Juckreiz bereits in der Woche nach der IA signifikant von 10,0 (Median) auf 0 (p = 0,0156).

In der vorliegenden Kohorte hatten alle Patienten mindestens ein UE (insgesamt 56 UE), was höher ist als in der Studie von Hübner et al., die nur bei 65% der Patienten mindestens eine UE beschrieb. Bei zehn der 56 UE in der vorliegenden Studie handelte es sich um Anämie/Dyspnoe, eine häufige Nebenwirkung von Dapson. Bei drei von fünf Patienten musste Dapson dauerhaft abgesetzt werden, während bei den beiden anderen eine Dosisreduzierung von Dapson zu einem Anstieg des Hb führte. Insgesamt wurden 15 UE bei fünf Patienten als schwerwiegend (Grad 3 oder 4) eingestuft, von denen acht möglicherweise mit dem Studienmedikament zusammenhingen, während bei sieben ein Zusammenhang unwahrscheinlich erschien.

Das mittlere Intervall zwischen dem letzten Tag der IA und dem Auftreten der schweren UE betrug 84 Tage, wobei die früheste UE 26 Tage nach der IA auftrat. Schwerwiegende UE, die möglicherweise mit der IA zusammenhängen, waren Angina pectoris, Ekzem, BP‐Rezidiv, hypertensive Krise und unklare Infektion bei vier Patienten, die jeweils 35, 28, 26, 50, 71, 223, 28 und 81 Tage nach der letzten IA auftraten. Insgesamt kann nicht ausgeschlossen werden, dass einige schwerwiegende UE mit der IA in Zusammenhang stehen. Die große zeitliche Latenz zwischen ihrem Auftreten spricht unserer Meinung nach nicht für einen kausalen Zusammenhang. Bemerkenswert ist, dass mit Ausnahme einer unklaren Infektion bei Patient 3, 12 Wochen nach der letzten IA keine systemischen Infektionen auftraten. Infektionen wurden bereits in früheren Fallberichten und Studien mit IA in Verbindung gebracht, meist bei Patienten mit zentralem Venenzugang.^27^ In der vorliegenden Studie wurde bei allen Patienten ein zentraler Zugang gelegt.

Bemerkenswert ist, dass die meisten schweren UE (46%) bei einem einzigen Patienten auftraten. Dieser Patient wies mehrere Komorbiditäten auf, darunter eine koronare Herzkrankheit (die zum Zeitpunkt des Studieneinschlusses nicht als schwere kardiovaskuläre Erkrankung galt, da sie asymptomatisch war) und Nierenversagen, und war 10 Jahre älter als das Durchschnittsalter der Kohorte. Bislang wurde ein höheres Alter noch nicht mit einer höheren Rate schwerer Nebenwirkungen bei Patienten die mit IA behandelt werden in Verbindung gebracht.^22^, ^36^

Tatsächlich berichteten Hübner et al. über eine 94‐jährige Frau mit BP, die als einzige UE eine Anämie entwickelte. Interessant ist, dass keiner unserer Patienten während des Nachbeobachtungszeitraums von 1 Jahr verstarb, obwohl die Ein‐Jahres‐Mortalität bei BP‐Patienten 20–30% beträgt und im Vergleich zu geschlechts‐ und altersgleichen Kontrollen etwa 3–5‐mal höher ist.^37^, ^38^, ^39^ Man könnte spekulieren, dass die niedrige Sterblichkeit in unserer Kohorte auf das potenziell kortikosteroidsparende Schema zurückzuführen ist, das ein schnelleres Absetzen von Prednisolon ermöglicht. Diese Hypothese wird durch die Beobachtung einer Ein‐Jahres‐Mortalität von 16% in einer multizentrischen Beobachtungsstudie mit 198 BP‐Patienten gestützt, die ausschließlich Prednisolon in einer Anfangsdosis von 0,5 mg/kg KG/Tag erhielten.^40^

Bei allen unseren Patienten sanken die Serumspiegel der IgG‐Autoantikörper gegen BP180 nach der IA signifikant, und zwar um durchschnittlich 85% gegenüber dem Ausgangswert unmittelbar nach der dritten IA und um 68% 4 Wochen später. Dies ist vergleichbar mit der Fallserie von Hübner et al., die einen anfänglichen Rückgang der Anti‐BP180‐IgG‐Antikörper um 92% unmittelbar nach der dritten IA‐Behandlung und um 74% 4 Wochen später beobachteten. Insgesamt stellen wir die Hypothese auf, dass die IA durch die rasche Senkung der zirkulierenden Autoantikörper dazu beiträgt, die Krankheitsaktivität in der frühen Behandlungsphase zu verringern, was anschließend ein schnelleres Absetzen der Kortikosteroide ermöglicht. Angesichts der schweren Nebenwirkungen von Kortikosteroiden ist dies ein großer Vorteil für unsere Patienten.

Eine Limitation unserer Studie ist das Fehlen einer Kontrollgruppe, was eine viel größere Studiengröße von etwa 100 Patienten bedeutet hätte, wie sie zuvor in der IA‐Pem‐Studie für Pemphigus geschätzt wurde.^27^ Eine weitere Limitation ist die Verwendung der IA als adjuvante Behandlung in Kombination mit einer Therapie, die in nationalen und internationalen Leitlinien für mittelschweres/schweres BP empfohlen wird.^14^, ^41^

Zusammenfassend lässt sich sagen, dass unsere prospektive, nicht kontrollierte monozentrische Studie gezeigt hat, dass die IA eine relativ sichere und potenziell wirksame adjuvante Therapie für Patienten mit schwerem und/oder refraktärem BP ist. Da es unwahrscheinlich ist, dass eine randomisierte kontrollierte Studie durchgeführt wird, die hoch evidente Informationen über den Wert der IA bei BP liefern würde, stellen die hier vorgestellten aktuellen Daten möglicherweise die höchstmögliche Evidenz für die Sicherheit und Wirksamkeit der IA bei dieser Erkrankung dar.

## DANKSAGUNG

Wir danken Dana Schmidt für die exzellente technische Hilfe.

Open access Veröffentlichung ermöglicht und organisiert durch Projekt DEAL.

## FINANZIERUNG

Die Arbeit wurde unterstützt von Fresenius Medical Care.

## INTERESSENKONFLIKT

E.S. hat Honorare für Vorträge und Reisekosten von Fresenius Medical Care erhalten. ES hat eine Forschungskooperation mit Fresenius Medical Care. N.v.B. hat Honorare für Vorträge von Fresenius Medical Care erhalten.

## Supporting information



Supplementary information
